# Supplementary material for: Genome-Wide Identification of ARF Gene Family Suggests a Functional Expression Pattern during Fruitlet Abscission in Prunus avium L
Source: Int J Mol Sci. 2021 Nov 4;22(21):11968. doi: 10.3390/ijms222111968 (PMC8584427; doi:10.3390/ijms222111968)
Supplement: Supplementary file 1 [file ijms-22-11968-s001.zip › Table S1.pdf]

Table S1. Primesr used for qRT-PCR of PavARFs from *Prunus avium*.

Gene	Sequence (5'-3')	Length (bp)	TM (°C)	Product length (bp)	Temperature (°C)
PavARF1-F	GTTTCTCTGTGCCCCGTCGTG	21	61.8	283	60.5
PavARF1-R	GGGTTGGTGGGTTGCCTGTTAG	22	61.5		
PavARF2-F	CGTTCCCATCCTCCATTCTA	20	57.3	283	58.5
PavARF2-R	GCCGAACGAAACGACCTTAT	20	59.4		
PavARF3-F	CCTCTCACCCATTACGAACAAA	22	59.4	257	58.5
PavARF3-R	TCAAGCACGACTTCTCAGCATC	22	60.4		
PavARF4-F	CGTCCAAGCAACTCCAACTCA	21	61.0	180	59.5
PavARF4-R	CCATCACCCCTCAAAAACACA	21	60.5		
PavARF5-F	CCACATCATTTGCCAAGACATT	22	59.9	135	57
PavARF5-R	CAAGAATGGTCTGAACAGGAGG	22	58.8		
PavARF6-F	GGGTCTTTCCTCCTCTTGATTAC	23	58.3	250	58
PavARF6-R	CCAGTCTGTGGACGATTTGC	20	58.5		
PavARF7-F	TCGGTGGTTTAGGTGGTCAG	20	58.1	129	57
PavARF7-R	AGGTAATCTCAGAGTCCGCAAG	22	58.1		
PavARF8-F	CTTCCTCCTTTGGATTTCGC	20	57.7	247	56
PavARF8-R	GGTGGAAAGAGGGTAGTCCAG	21	57.8		
PavARF9-F	GCCAGATTACTTTACTACCCGACAC	25	60.9	389	58
PavARF9-R	CACTCCAACACGCAACTCCC	20	60.9		
PavARF10-F	GACCATACCTCGTCCAGACA	20	55.1	206	57
PavARF10-R	AGACCCTTGAAAACCCACC	19	56.0		
PavARF11-F	GGGACTATAAGCAGCAGAGGC	21	58.5	179	58
PavARF11-R	GCGAGAAAGAACACTGGGATAA	22	58.9		
PavARF12-F	CGTCCAGACCGTGATTGCTAA	21	61.0	197	60
PavARF12-R	CGGCTCGCCTAATCCCAAC	19	62.8		
PavARF13-F	CCCCTTTGGTTGTCTGCCCGATAA	24	65.0	158	60
PavARF13-R	GAGTACGAGCTGCGGTGGCTTTA	23	65.7		
PavARF14-F	GCGAGACCTCCAGAACTTACT	21	55.5	139	57.5
PavARF14-R	CGCATCACTTGAGCACCC	18	57.4		
PavARF15-F	GCGGGTTTCCTAACTTGGC	19	59.7	155	57.5
PavARF15-R	GCGAAACTGCGGTAGTAATGG	21	60.2		
PavARF16-F	GCCAACCCAAACGACATCTT	20	59.8	358	58.5
PavARF16-R	CGCTCAAATAGCATCCGAAAT	21	59.8		
