# Supplementary figures and images for: Genome-Wide Identification of ARF Gene Family Suggests a Functional Expression Pattern during Fruitlet Abscission in Prunus avium L
Source: Int J Mol Sci. 2021 Nov 4;22(21):11968. doi: 10.3390/ijms222111968 (PMC8584427; doi:10.3390/ijms222111968)

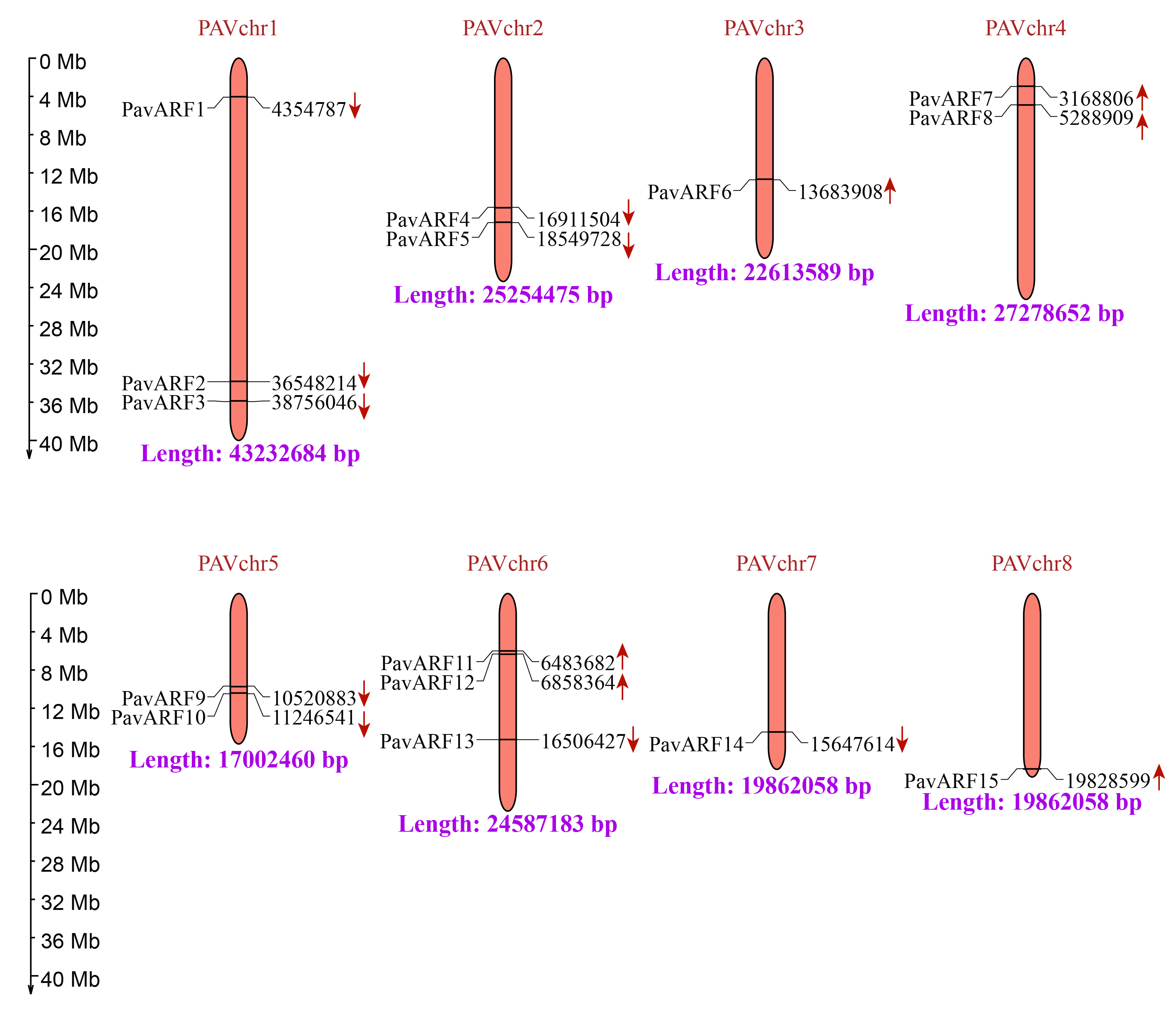

Supplement: Supplementary file 1 [file ijms-22-11968-s001.zip › Figure S2.tif]
